# Intravenous Leiomyomatosis of the Uterus: A Retrospective Single-Center Study in 14 Cases

**DOI:** 10.1155/2020/9758302

**Published:** 2020-02-14

**Authors:** Qingbo Su, Xiquan Zhang, Hui Zhang, Yan Liu, Zhaoru Dong, Guangzhen Li, Xiangjiu Ding, Yang Liu, Jianjun Jiang

**Affiliations:** ^1^Department of Vascular Surgery, Qilu Hospital of Shandong University, Jinan, Shandong 250012, China; ^2^Department of Cardiac Surgery, Qilu Hospital of Shandong University, Jinan, Shandong 250012, China; ^3^Department of Gynaecology, Qilu Hospital of Shandong University, Jinan, Shandong 250012, China; ^4^Department of Cardiology, Qilu Hospital of Shandong University, Jinan, Shandong 250012, China

## Abstract

**Purpose:**

This study aimed to retrospectively review the diagnosis and surgical treatment of uterine intravenous leiomyomatosis (IVL).

**Methods:**

The clinical data of 14 patients with uterine IVL admitted to our hospital between 2013 and 2018 were retrospectively analyzed, including their demographics, imaging results, surgical procedures, perioperative complications, and follow-up results.

**Results:**

The tumors were confined to the pelvic cavity in 7 patients, 1 into the inferior vena cava, 4 into the right atrium, and 2 into the pulmonary artery (including 1 into the superior vena cava). Only one case was misdiagnosed as right atrial myxoma before the operation, which was found during the surgery and was treated by staging surgery; all the other patients underwent one-stage surgical resection. Three patients underwent complete resection of the right atrial tumor through the abdominal incision, and one patient died of heart failure in the process of resection of heart tumor without abdominal surgery. During the 6–60 months of follow-up, 4 patients developed deep venous thrombosis of the lower extremity, and 1 patient developed ovarian vein thrombosis and pulmonary embolism. After anticoagulation treatment, the symptoms disappeared. One patient refused hysterectomy and the uterine fibroids recurred 4 years after the operation.

**Conclusion:**

Specific surgical plans for uterine IVL can be formulated according to cardiac ultrasound and computed tomography (CT). For the first type of tumor involving the right atrium, the right atrium tumor can be completely removed through the abdominal incision alone to avoid thoracotomy. The disease is at high risk of thrombosis and perioperative routine anticoagulation is required.

## 1. Introduction

Uterine intravenous leiomyomatosis (IVL) is an uncommon uterine smooth muscle tumor which is defined as an intraluminal growth and extension of benign smooth muscle from the intrauterine venules upward to varying distances, including the pelvic veins, the inferior vena cava, the right atrium, and pulmonary artery [1, 2]. Early diagnosis of this disease is difficult owing to its insidious onset and when symptoms appear, the tumor is often involved in the right atrium and even the pulmonary artery, causing serious consequences [3]. Complete surgical resection is currently the most effective intervention for patients with IVL of the uterus [4, 5]; however, the surgeries are all carried out under deep hypothermia circulation arrest combined with thoracic and abdominal incision and circulatory anesthesia, resulting in huge surgical trauma.

The specific etiology of IVL is not yet clear, and there are two theories: one is that the leiomyomas originate from the uterine vein wall; second, invasion of uterine fibroids into blood vessels [6]. In the past, simple IVL without uterine fibroids has been rarely reported, and most scholars are inclined to the second view at present [7]. Birsh-Hirschfeld first described IVL in 1896 [8]. To date, fewer than 400 cases of uterine IVL had been reported in global English literature. Our study comprised 14 patients with uterine IVL from our hospital between 2013 and 2018, among which 7 cases involved inferior vena cava/heart/pulmonary artery. Besides, we further simplified the surgery based on previous studies, and there were 3 cases of complete pelvic, inferior vena cava, and right atrium tumor resection through a single abdominal incision. Our work may contribute to making a surgical plan.

## 2. Patients and Methods

### 2.1. Patients

This study was conducted with the approval of the Qilu Hospital of Shandong University Research Ethics. Committee and written informed consent was obtained from the patients participating in this study. Fourteen female uterine IVL cases (age range: 33–66 years, mean age: 46 years) were recruited from Qilu Hospital of Shandong University between 2013 and 2018 and were analyzed retrospectively. Gynecological ultrasound and echocardiography were performed for all patients, and an enhanced computed tomography (CT) examination was performed for patients with uterine IVL involving inferior vena cava or heart (right atrium). Among the 7 patients involved in inferior vena cava or heart, 3 had previous uterine fibroid surgery, 1 underwent right atrial tumor resection, and 1 had a history of uterine fibroids for 11 years without treatment. The maximum diameter of premenopausal uterine fibroids was 11 cm, and the tumor shrank to 8 cm from postmenopausal to preoperative physical examination.

To better guide surgical strategies and standardize the operation, the classification method of Ma et al. with modifications was applied to divide uterine IVL into four stages according to the disease progression [5]: stage I: lesions were confined to the pelvis; stage II: the lesion was located in iliac vein/inferior vena cava; stage III: tumor entered the right atrium/right ventricle; stage IV: the tumor reached the pulmonary artery. There were 7 patients with stage I, including 1 patient with the lower abdominal bulge, 2 patients with increased menstruation, 1 patient with dysmenorrhea, and the remaining 3 patients with no symptoms who were checked with uterine fibroids at physical examination. One case was in stage II, who presented dysuria and left lower extremity edema. There were 4 patients in stage III, including 1 patient with chest stuffiness, 1 patient with abdominal pain, 1 patient with recurrent syncope, and 1 patient with no symptoms (due to the recurrence of uterine fibroids after surgery, the tumor in the right atrium was found on admission for physical examination). Stage IV included two patients: one presenting with fatigue and recurrent syncope and the other with chest stuffiness (orthopnea). All intracardiac leiomyomatosis (ICL) patients had different degrees of symptoms of cardiac dysfunction (Supplementary Table 1). One patient was in the New York Heart Association (NYHA) functional class IV, three patients were in NYHA functional class III, and two were in NYHA functional class II.

### 2.2. Surgical Approach

All the 7 patients at stage I were diagnosed as uterine leiomyoma preoperatively, and lesions in 2 patients were confined to the uterus and underwent hysteroscopic myomectomy. Postoperative pathology confirmed the disease. Five cases are intraoperatively found with a funicular neoplasm within the blood vessels of the broad ligament of the uterus. Considering this disease, we further confirmed it by intraoperative rapid pathologic examination and then the uterus and bilateral attachments including the tumor were removed. In Case 8, the uterus was found to be about 4-month gestation size during the operation, with multiple tumors, the largest of which was 13 cm in diameter. In the process of tumor resection, a cordlike tissue was found in the parametrial blood vessels, highly suspected of IVL, which was confirmed by rapid pathology during the operation. The uterus and bilateral attachment resection were recommended, but the patient's family requested to preserve the reproductive function and refused to remove the uterus and ovaries; thus, simple hysteromyoma resection was performed.

### 2.3. Operative Procedure

**Figure 1:**
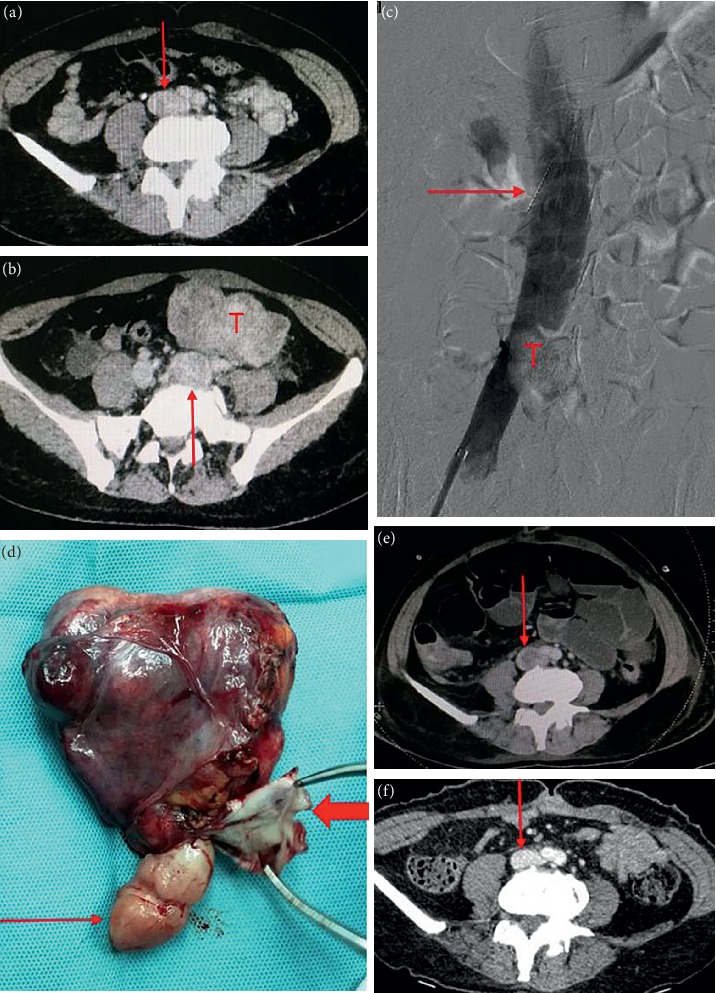
Case 1. (a) Enhanced CT shows uneven intensity in the inferior vena cava (arrow). (b) Marked dilation of the left common iliac vein (arrow), with the same density as pelvic tumor (T). (c) Angiography shows the filling defect of the inferior vena cava (T), with arrow indicating the filter of the inferior vena cava. (d) The tumor specimen. Thin arrow indicates the tumor in the inferior vena cava, and thick arrow indicates the wall of the left internal iliac vein. (e) CT reexamination at 1 month after surgery shows thrombus formation at the beginning of inferior vena cava (arrow). (F) CT shows thrombus disappearance of inferior vena cava at 2 months after anticoagulation.

**Figure 2:**
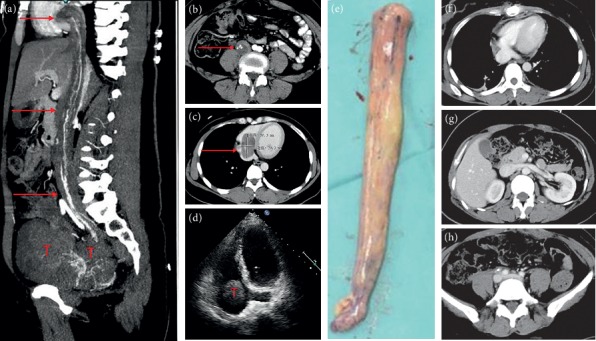
Case 2. (a, b) CT at arterial phase showed tumors in the right iliac vein, inferior vena cava, and right atrium, and arterial blood supply could be seen within the tumor. (c) Venous phase CT; tumor size = 5 *∗* 3 cm in the right atrium. (d) Cardiac ultrasound showed the tumor in the right atrium with clear boundaries, high activity, and no adhesion with the atrium. (e) Tumor specimens of iliac vein/inferior vena cava/right atrium. (f, g, h) CT at three months after the operation. The right heart, inferior vena cava, and iliac vein were unobstructed.

**Figure 3:**
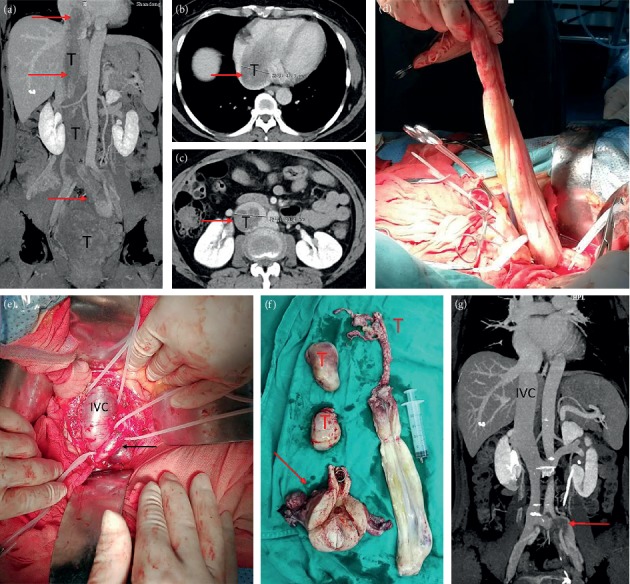
Case 3. (a, b, c) Tumor entered the right atrium from the right iliac vein through the inferior vena cava, and the tumor diameter in the right atrium was 4 cm. (d) The tumor was pulled out from the inferior vena cava. (e) The arrow shows the left common iliac vein compressed by the right common iliac artery. (f) The arrow points to the resected uterus and bilateral adnexa. (g) Postoperative CT shows unobstructed right atrium and inferior vena cava, and the arrow indicates left common iliac vein thrombosis.

**Figure 4:**
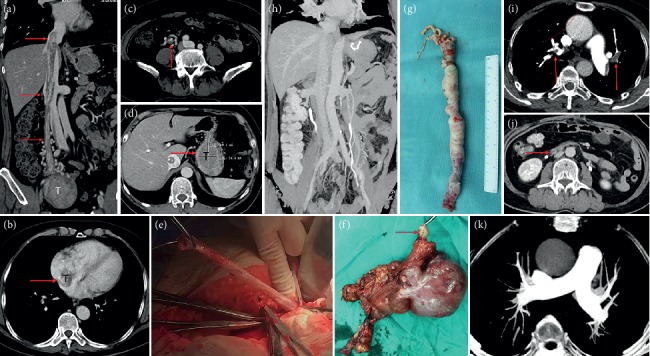
Case 4. (a) CT shows that the tumor enters into the inferior vena cava/right atrium through the right ovarian vein. (b) Right atrial tumor. (c) Arrow shows ovarian intravenous filling defect. (d) Intragastric tumor (leiomyoma was considered preoperatively); (e) the tumor is pulled out from the right ovarian vein. (f) Uterus and bilateral attachments, multiple tumors protrude from the uterine wall and the largest is 8 cm in diameter; the arrow refers to the ovarian intravenous tumor; (g) ovarian vein-inferior vena cava-right atrial tumor; (h) postoperative CT examination reveals unobstructed inferior vena cava. (i) CT shows bilateral pulmonary embolism at 10 days after surgery. (j) Right ovarian vein stump is filled with thrombus (arrow). (k) CT at 3 months after surgery shows that pulmonary artery thrombosis disappeared.

All patients underwent surgery in the supine position under general anesthesia. Of the patients in stage I, 3 patients were treated with hysteromyoma resection alone, and the remaining patients were treated with pelvic tumor resection and hysterectomy as well as bilateral adnexectomy. For Case 1, to prevent pulmonary embolism caused by tumor shedding, the inferior vena cava filter (William Cook Europe, Bjaeverskov, Denmark) was implanted under local anesthesia, and tumor resection was performed 3 days later. During the operation, the inferior vena cava and left common iliac, internal and external iliac veins were dissociated. It was planned to block the inferior vena cava and external iliac vein and the internal iliac vein was cut open to remove the tumor. However, during the tumor removal process, the pelvic tumor was directly removed from the left internal iliac vein together with the inferior vena cava tumor (Figure 1), not blocking the inferior vena cava. Case 2 was the first case of intracardiac leiomyomatosis (ICL). We planned to perform a primary surgical resection. First, we took a ventral midline incision. Pelvic tumor and uterus + bilateral attachments were firstly removed by the gynecological surgeon. Then, the heart surgery took the sternal midline incision to establish extracorporeal circulation. After cardiac arrest, the right atrium was opened, and no tumor was found, suggesting that the tumor probably retracted into the inferior vena cava. Finally, the tumor was completely removed by vascular surgery through the right iliac vein (Figure 2). We found that the tumor had no adhesion to the inferior vena cava; however, it adhered tightly to the right internal iliac vein. After undergoing these two surgeries and reviewing the literature [9–12], we found that most of the tumors were free in the inferior vena cava and the right atrium. It was assumed that the tumors in the right atrium could be removed directly through abdominal incision, avoiding thoracotomy to establish extracorporeal circulation. For the next two patients (Case 3 and Case 4), we prepared extracorporeal circulation and temporarily did not perform thoracotomy. The pelvic tumor, uterus, and bilateral adnexa were resected through the mid-abdominal incision. In Case 3, the inferior vena cava was cut open and the proximal tumor was pulled out from the right atrium (Figure 3). In Case 4, the tumor entered into the inferior vena cava through the right ovarian vein. Because the tumor was located in the inferior vena cava above the entrance of the ovarian vein, if the tumor was removed from the inferior vena cava, the liver should be lifted up to free the inferior vena cava above the renal vein. Due to the obvious expansion of the right ovarian vein, and to further reduce the surgical trauma, we cut open the ovarian vein and completely removed the tumor in the right atrium/inferior vena cava (Figure 4). Then the stomach tumor was removed by gastrointestinal surgery, and the tumor and stomach wall were removed together along the edge of the tumor by 1 cm. During the operation, cardiac ultrasound examination was performed to confirm that there was no residual intracardiac tumor. The cardiac surgeons prepared the extracorporeal circulation. If the tumor cannot be removed, thoracotomy should be performed immediately through an incision in the right atrium.

**Figure 5:**
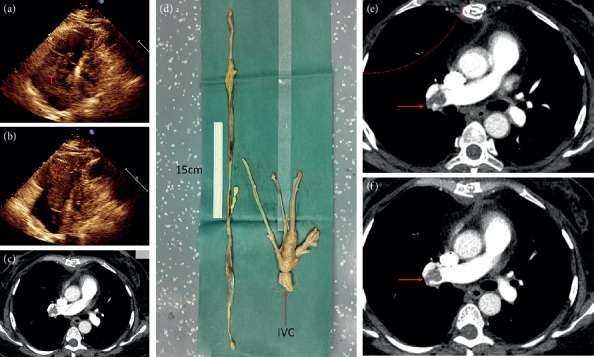
First-stage surgery for case 5. (a) Cardiac ultrasound shows systolic tumor located in the right atrium. (b) Diastolic tumor into the right ventricle. (c) Right pulmonary embolism. (d) Right atrium, right ventricle, and pulmonary artery tumor. Arrow refers to the cut off site of inferior vena cava and the length of the tumor in the heart is more than 70 cm. (e, f) CT reexamination at 3 and 6 months after surgery showed no change in right pulmonary embolism compared with that before surgery.

**Figure 6:**
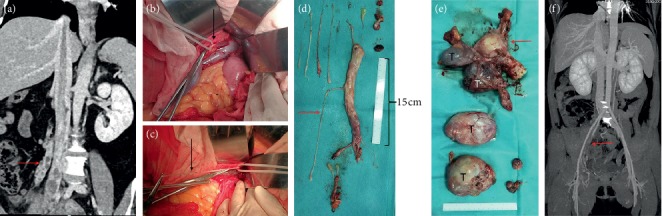
Second-stage surgery for case 5. (a) The tumor enters the inferior vena cava from the right iliac vein and the right ovarian vein (arrow). (b) The right ovarian vein is visible through the venous vein wall. (c) The tumor is pulled out from the right ovarian vein. (d) Tumors in the inferior vena cava/iliac vein/ovarian vein. (e) Pelvic tumor. Arrow refers to the uterus and bilateral attachment; (f) CT scan at 6 months after surgery shows unobstructed inferior vena cava and thrombosis of right iliac vein (arrow).

Case 5 was misdiagnosed as myxoma of the right atrium before the surgery and underwent staging surgery. According to the preoperative echocardiography, Case 5 was considered as right atrial myxoma combined with right pulmonary embolism (Figure 5). A chest midline incision was made and extracorporeal circulation was established. After cardiac arrest, the right atrium was opened. We found that the tumor was originated from the inferior vena cava, in a free state in the right atrium, extending into the right ventricle and pulmonary artery, and adhering to the right ventricle. Then, we considered it as the IVL. Because abdominal and gynecological imaging examinations were not performed preoperatively, staging surgery was decided for Case 5, and the tumor within the heart and pulmonary artery was firstly removed (Figure 5). The operation was successful and she was discharged 12 days later. One month after the surgery, the right lower extremity edema suddenly occurred. Ultrasound examination showed thrombosis of the right iliofemoral and popliteal vein, and the patient was readmission to hospital. Cardiac color Doppler ultrasound showed that the tumor was located at the atrial entrance of the inferior vena cava, and no tumor was found in the cardiac chambers. CT showed multiple tumors in the uterus, which entered the inferior vena cava through the right iliac vein and the right ovarian vein, respectively (Figure 6). Laparotomy was performed for pelvic tumor and uterus resection as well as bilateral adnexectomy. Then, the right ovarian vein was cut open to pull the tumor out and ligated close to the inferior vena cava, followed by cutting off the right ovarian vein. Finally, the inferior vena cava was dissected, and the tumor at the proximal end of the heart rushed out of the incision by itself along with the blood flow. The distal end was fixed in the internal iliac vein. After the internal iliac vein was cut open, the tumor was removed and the internal iliac vein was ligated. The right external iliac vein was dissected and the thrombus of right iliac vein and femoral vein was removed.

**Figure 7:**
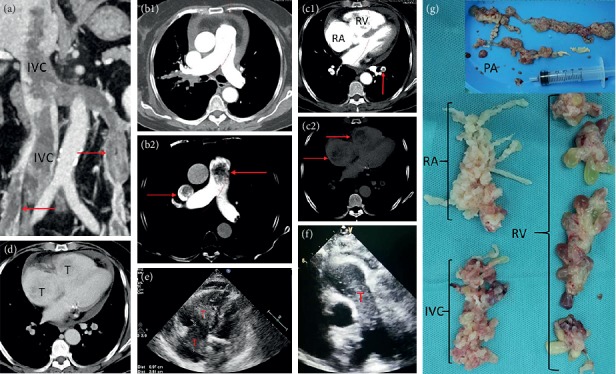
Case 6. (a) CT shows that the tumor enters the inferior vena cava from the right iliac vein and bilateral ovarian veins (arrows). The tumor in the right ovarian vein directly enters the inferior vena cava, and the tumor in the left ovarian vein enters the inferior vena cava via the left renal vein. (b1) and (b2) are the same image. (b1) shows that the main pulmonary artery and the superior vena cava are unobstructed under conventional CT brightness. (b2) can show the low-density shadow in the main pulmonary artery and the superior vena cava after adjusting the contrast (arrow). (c1) and (c2) were the same image in the arterial phase. The arrow of (c1) showed left inferior pulmonary artery embolism, and no obvious abnormalities were found in the right atrium and right ventricle. Low-density shadows in the right atrium and right ventricle were faintly visible after adjusting the contrast of (c2) (arrow). (d) The venous stage. The right atrium and right ventricular tumor can be faintly seen. (e) Cardiac ultrasound showed right atrium and right ventricular tumor with unclear boundary, (f) echocardiography shows intrapulmonary tumors. (g) A part of the removed tumor specimens, with irregular pattern (cauliflower-like), brittle texture, and easy fracture.

Case 6 was at stage IV with cardiac function at grade IV and had complete obstruction of the main pulmonary artery and bilateral pulmonary embolism (Figure 7). It was proposed to do thoracoabdominal incision and resection of the tumor for one-stage surgery. However, after anesthesia, the patient's blood pressure suddenly dropped and blood oxygenation was undetectable. Moreover, considering cardiogenic shock, emergent thoracotomy was performed and extracorporeal circulation was established. Right atrium was cut open and the tumors in the right atrium, right ventricle, and inferior vena cava at the right atrium entrance were removed. During the operation, the tumor was found to enter the superior vena cava, adhered to the superior vena cava and the tumor was resected from the superior vena cava. The main pulmonary artery was opened and we found that the tumor occupied the main pulmonary artery and entered the bilateral pulmonary artery. The visible tumors in the main pulmonary artery and branch were taken out. Because the heart could not resume beating, the patient died during the operation, so no abdominal surgery was performed.

### 2.4. Histopathology Analysis

All tumor specimens were sent for pathological examination. Specimens were fixed with standard formalin and sectioned into multiple blocks. Pathological sections were stained with hematoxylin and eosin and examined by a pathologist.

## 3. Results

### 3.1. Clinical Details

Among the 7 patients with lesions involving inferior vena cava and heart, except Case 5 which was misdiagnosed as right atrial myxoma before surgery, the other 6 cases were all diagnosed as uterine IVL before surgery. The tumor in Case 1 grew along the left iliac vein, extending to the inferior vena cava below the level of the renal veins. The tumor in Case 2 and Case 7 grew through right internal iliac vein-common iliac-inferior vena cava to right atrium. The tumor in Case 3 grew through the left iliac vein-inferior vena cava to the right atrium; the tumor in Case 4 entered into the right atrium through the right ovarian vein and inferior vena cava. The tumor in Case 5 passed through the right iliac vein and the right ovarian vein-inferior vena cava to the right heart and involved the pulmonary artery. The tumor in Case 6 passed through the inferior vena cava via the right iliac vein and bilateral ovarian veins and then entered into the right atrium, right ventricle and pulmonary artery. The patient characteristics are shown in Table 1.

All the 14 patients underwent surgery (Case 7 was diagnosed with uterine IVL in our hospital and went to other hospitals for surgical treatment. Case 7 survived without discomfort till now). One patient died during the operation, and no patient died after the operation. The disease was not diagnosed preoperatively in all stage I patients and was confirmed by rapid intraoperative pathology in 5 patients. The lesion was located in uterus in 2 patients, and IVL was confirmed by postoperative pathology. For case 8, uterine and ovary preservation was strongly required due to the requirement of preserving fertility function, and only simple hysteromyoma resection was performed. Four years after the surgery, uterine fibroids recurred, but the patient still did not agree to reoperation and was still in the follow-up. For Case 1, postoperative pelvic and wound infection occurred, and the hospital stay was more than 1 month. Case 5 was combined with gastric neoplasm, which was considered as gastric leiomyoma before surgery and confirmed as gastric stromal tumor by postoperative pathology. In Case 6, cardiac function was at grade IV. This patient was applied for orthopnea. Due to patients staying in bed for a long time, 1 day before surgery, extensive thrombosis of the left lower extremity deep vein occurred; the patient died of heart failure during surgery.

In all patients with intrainferior vena cava tumor, anticoagulation began 12–24 hours after surgery and continued until discharge. Patients with thrombosis continued to take oral anticoagulant for 6 months. For Case 2, therapy with subcutaneous low molecular weight heparin (5000 u ih qd) and oral warfarin 3.75 mg per day was started after surgery, and the PT-INR values maintained between 2 and 3. The rest of the thrombus patients were given rivaroxaban 20 mg qd orally.  Table 2 showed the clinical data of patients with intrainferior vena cava tumor. The Caprini risk assessment model was used to forecast preoperative thrombosis.

### 3.2. Pathologic Findings

**Figure 8:**
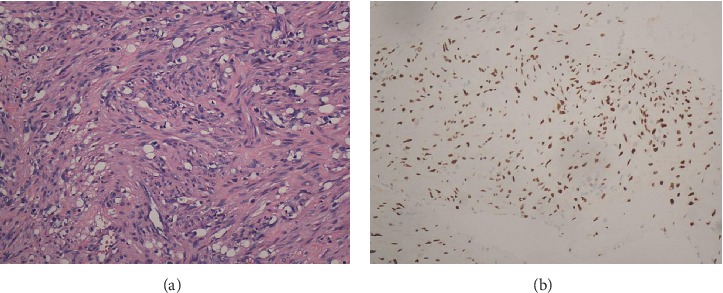
Histopathology of intracardiac leiomyomatosis (ICL). (a) Typical smooth muscle cells. The spindle cells are arranged in a spiral shape with no obvious atypia (×200); (b) the ICL tumor is positive for oestrogen receptor (×200).

All patients were diagnosed with uterine IVL by postoperative pathology. Immunohistochemical staining was positive for smooth muscle markers (desmin and smooth muscle actin). Oestrogen receptor (ER) and progesterone receptor (PR) were expressed to varying degrees. These morphological and immunohistochemical findings have been consistent with typical IVL findings (Figure 8 and Supplementary Table 1).

### 3.3. Follow-Up Analysis

The patients were followed up at 1, 3, and 6 months after discharge and then followed up annually. For patients with stage I, only pelvic ultrasound examination was performed. For patients with stage II and above, enhanced CT and cardiac ultrasound examination were carried out. The follow-up period was 6–60 months (average: 25 months). Except for one intraoperative death, 13 patients survived during the follow-up without serious complications. There were 5 cases of venous thrombosis, including 4 cases of deep venous thrombosis (DVT) of lower extremity and 1 case of right ovarian vein venous stump thrombosis and pulmonary embolism (Figure 4). They were stage II, III, and IV patients; all of whom were treated with rivaroxaban orally for anticoagulation. Case 6 occurred DVT 1 day before surgery, and the remaining 4 cases occurred DVT postoperatively. For Case 1, postoperative follow-up by enhanced CT examination revealed a thrombus at the beginning of the left common iliac vein-inferior vena cava. After 1 month of anticoagulation, the thrombosis of the inferior vena cava disappeared and the thrombosis of the left common iliac vena cava narrowed. After removing the filter of the inferior vena cava, the anticoagulation was continued for 6 months. Case 3 had left common venous thrombosis and no symptoms of lower extremity edema, which was found by CT examination in postoperative follow-up. Case 4 was found by CT reexamination 1 month after surgery, without symptoms of chest distress, and pulmonary embolism and ovarian venous thrombosis disappeared 3 months after anticoagulation (Figure 4).

Case 5 was a patient undergoing staged surgery. One month after cardiac surgery, the DVT of the right lower extremity suddenly occurred. The right iliofemoral vein thrombectomy was performed at the same time during the second-stage surgery. The right lower extremity edema was relieved after surgery. The CT examination after 6 months of anticoagulation found that there was still a thrombus in the right iliac vein and the right femoral vein was unobstructed. Compared with the preoperative period, the two CT examinations after right pulmonary embolism operation showed no change. Case 8 had a recurrence of uterine fibroids at 4 years after surgery, and the patient did not undergo reoperation and was under continuous observation. All the 6 patients with IVL involving the heart had improved cardiac function except 1 patient who died during the operation. Besides, 3 patients returned to normal and 2 patients had cardiac function at grade II.

## 4. Discussion

Uterine leiomyoma is the most common tumor of the female reproductive system. Among women of reproductive age, the incidence rate of uterine leiomyoma is up to 43% in white and 59% in black people [13]. Uterine IVL is a special type of uterine leiomyoma, which is extremely rare in patients with cardiac involvement. All of the 14 patients we reported to have a history of hysteromyoma or hysteromyoma resection, and the results supported the view that uterine IVL originates from invasion of uterine fibroids into blood vessels [6].

Uterine leiomyomas can grow along the veins or lymphatic vessels, but there are two main ways to grow up; one is through the uterine vein—internal iliac vein—common iliac vein—inferior vena cava to reach the right atrium, right ventricle, and even the pulmonary artery; another route is through the right ovarian vein (left ovarian vein—left renal vein)—inferior vena cava to reach right cardiac chambers [14]. Among the 7 patients involved in the inferior vena cava, 4 patients' tumors grew through the first route (1 patient on the left side, 3 patients on the right side), and only 1 patient's tumor through only the second route (right ovarian vein). The tumor in the two patients grew at the same time through two ways. In Case 6, the tumor entered the inferior vena cava through the right uterine vein and bilateral ovarian vein. It was found that the tumor in Case 6 occupied the right atrium, right ventricle, and the main pulmonary artery, reached the bilateral pulmonary artery, and entered the superior vena cava through the right atrium. However, preoperative cardiac ultrasound and CT showed no lesions in the superior vena cava. After the surgery, we carefully reviewed the preoperative CT and found tumors in the superior vena cava after adjusting the contrast (Figure 7(b)). To the best of our knowledge, this is the first time that IVL involving the superior vena cava was reported.

Of the 14 patients, the tumors of only two (14.3%) patients were confined to the uterus, and 12 cases (85.7%) had lesions that reached the extrauterine blood vessels (the tumor in 5 cases invaded the vessels of the broad ligament, in 1 case it entered the inferior vena cava, in 4 cases it entered the right heart, and in 2 cases it involved the pulmonary artery). Previous literature reported that 80% of IVL reached the extrauterine vessels [15], indicating that the biological characteristics of this disease were basically consistent between Chinese Han people and western people.

The clinical manifestations of patients with uterine IVL are nonspecific, and the early symptoms are similar to those of uterine fibroids, which can be manifested as pelvic pain, abdominal swelling, and abnormal vaginal bleeding [16]. There were 7 cases of stage I, among which 3 cases had no discomfort and were found by physical examination. Another four cases showed lower abdominal bulge, dysmenorrhea, and increased menstruation. One case of stage II patient presented with edema of the lower extremity of the affected side, while stage III and IV patients mostly presented with chest distress, palpitation, syncope, and other cardiopulmonary symptoms (Table 1). If not treated promptly, the patient may die due to heart failure or pulmonary embolism. The intraoperative death of Case 6 was considered to be caused by cardiogenic shock (preoperative cardiac function was at level IV). Although the inferior vena cava of stage III and IV patients was occupied by the tumor throughout the whole process, the blood flow from the inferior vena cava was basically passable. In addition, due to the slow growth of the tumor in the inferior vena cava, there is enough time to establish sufficient collateral circulation, so the lower limbs are mostly free from venous reflux disorder. Only 1 patient in this group was admitted to the hospital due to lower extremity edema.

Most ICL patients had a history of uterine fibroids or uterine surgery, and Li et al. reported 194 ICL patients, including 104 patients (53.6%) with previous hysterectomy/myomectomy [17]. In this study, 3 of the 7 patients with stage II or above had a history of uterine surgery, 2 of whom underwent hysterectomy, 1 underwent uterine fibroid resection; 1 had a history of uterine fibroids for more than 10 years. One patient received resection of the right atrial tumor without abdominal and pelvic examination in local hospital. One year later, the cardiac tumor recurred and came to our hospital for treatment. After comprehensive examination, uterine fibroids, iliac veins, and inferior vena cava tumors were found.

The onset of IVL is concealed, and the early lesions are confined to the pelvic cavity. Preoperative definite diagnosis could not be made by virtue of clinical symptoms and imaging analysis [18]. If broad ligament intravenous funicular neoplasm is found during surgery, the disease should be considered and a diagnosis can be made by rapid pathological examination during surgery. Once diagnosed, the uterus and bilateral attachments including the tumor should be removed. Besides, the ovary and uterine vein should be ligated as high as possible. When the lesion is confined to the uterus, the IVL is still difficult to be diagnosed during the surgery. Among the 7 stage I patients, 5 of them were found intraoperatively with intravenous funicular neoplasm of the broad ligament, all of which were confirmed by rapid pathology. The lesions of another 2 cases were confined to the uterus, and the disease was diagnosed by postoperative pathology.

On gross examination, we divided the tumor into four types according to Yang et al. [19]. Because the first and second types were similar and the surgical methods were the same, we combined them into one type and added a new type. Our specific classification was as follows: the first tumor type, such as cases 1–3 (Figures 1–3), has a smooth surface and a tough texture, like rubber, which is hard to break; the second type, such as Case 4 and Case 5 (Figures 4 and 5): the tumors in the inferior vena cava are of the same type as the first type, but after entering the heart, they lose the restriction of the inferior vena cava during tumor growth, and their shapes are extremely irregular; the third type, as shown in Figure 6, is thread-like; the fourth type of tumor, as shown in Figure 7, is cauliflower-like, highly irregular, brittle, and can be broken after a slight pull. Although the morphology and texture of these four types of tumors are completely different, there is no difference in pathological and immunohistochemical results (Figure 8 and Supplementary Table 1). The first type of tumors accounted for the vast majority. Both cardiac ultrasound and CT showed low-density tumors with clear boundaries (Figures 2 and 3). It is easy to be identified preoperatively, and the operation is the simplest, which can be directly pulled out from the iliac vein or the inferior vena cava through abdominal incision to avoid thoracotomy and extracorporeal circulation. The textures of the second and third types are the same as the first type. But, because of their small diameter, the tensile strength is weak. If the tumor is confirmed to have no adhesion to the right atrium, we could try to pull out from the inferior vena cava. If it encounters resistance, we should take it out via the heart stoutly by thoracotomy. The fourth type of tumor is cauliflower-like, and the inside of the tumor is surrounded by blood, so it shows uneven density and unclear boundary under ultrasound and CT. Because the tumor was brittle and fractured with slight traction, the tumor in the heart could not be removed from the inferior vena cava; thus, combined thoracic and abdominal incision was needed to remove the tumor. As shown in Figure 7, although the tumor was filled with right atrium, right ventricle, pulmonary artery, and grew up into the superior vena cava, it was not easy to be detected by CT, and the tumor can be faintly visible only by adjusting the contrast.

We believe that for ICL, enhanced CT and cardiac ultrasound are indispensable. Cardiac ultrasound can detect the presence or absence of tumor attachment points in the cardiac lumen. If the tumor is found to be free in the heart chamber, the atrial myxoma can be excluded (myxoma adheres to the cardiac chamber wall). The inferior vena cava/iliac vein CTA should be used to further confirm the diagnosis of ICL. We thought that CT can provide better information for ICL than MRI. CT can not only determine the number and size of uterine lesions and uterine fibroids but also determine whether the tumor enters the inferior vena cava through uterine vein or ovarian vein, as well as the involvement of right atrium, right ventricle and even pulmonary artery. Based on the MRI and CT, misdiagnosis can be minimized. In addition, CT can also determine whether the blood flow in the inferior vena cava, iliac vein, and femoral vein is unobstructed, thus providing the best venous reflux access for extracorporeal circulation.

IVL should be differentiated from right atrial myxoma, renal carcinoma, intravenous leiomyoma sarcoma, and DVT. Myxoma is the most common benign tumor in the right atrium. Previous literature reported that 29.5% of IVL patients were misdiagnosed as right atrial myxoma before surgery [17]. Myxoma adheres to the atrium and does not enter the inferior vena cava, while the IVL remain free in the right atrium. Enhanced CT can detect lesions in the uterus and the inferior vena cava, thus making a clear diagnosis. Case 5 was misdiagnosed as right atrial myxoma because no abdominal and pelvic examination was performed before surgery. Therefore, for young and middle-aged women with right heart system tumors, routine abdominal and pelvic CT examination is required, especially for those patients with a history of uterine fibroids. Renal cancer is the most common metastatic tumor in the right atrium [20]. The tumor can enter the right atrium through the renal vein-inferior vena cava, and the renal tumor can be detected by CT. Venous thrombosis is often acute, with low density and no enhancement on CT results. Intravenous leiomyosarcomas are rarer and are difficult to differentiate from IVL. The main difference is that leiomyosarcomas generally originate from the wall of the inferior vena cava and can also occur in men. The authors also encountered a patient with a retroperitoneal tumor that entered the inferior vena cava and reached the right atrium via the left renal vein (Supplementary Figure 1), which should also be differentiated from this disease.

Complete surgical resection of the tumor is the only current effective treatment. The entire uterus and bilateral attachments should be removed together with the complete removal of the tumor. Previous literature reports that radical resection has almost no recurrence [15], and partial resection has a 5-year recurrence rate of 33.3% [17]. In our study, three patients only underwent simple tumor resection and retained the uterus. One of them had tumor recurrence 4 years after surgery while twelve patients who underwent radical resection were followed up for 6–60 months without recurrence.

There are currently two surgical options for IVL: one-stage surgery and staged surgery, which have no difference in surgical complications and prognosis [21–24]. Timmis et al. first reported the successful resection of intracardiac leiomyoma in 1980, but the abdominal lesions were not treated [25]. The earliest radical surgery was reported by Ariza et al. in 1982 [26], which was carried out in stages, with the first stage of intracardiac tumor resection and the second stage of abdominal and pelvic tumor resection. In 1984, Gonzalez-Lavin et al. were the first to adapt one-stage surgery to complete tumor resection through chest and abdominal incision simultaneously [27]. All of the above operations were performed under deep hypothermic circulatory arrest. With the development of surgical techniques, the surgical trauma was getting smaller and smaller. In 2000, Wakiyama et al. completed the one-stage radical operation with circulatory arrest and normal temperature anesthesia [28]. We believe that, as long as the patient's physical condition allows, a combined thoracic and abdominal incision can be performed to complete the radical surgery. At present, the staging operation is mainly applicable to the following cases: (1) the general condition of the patient is poor, and it is difficult to tolerate the combined thoracic and abdominal incision; (2) the tumor and the vessel wall are widely adhered tightly; (3) in emergency surgery, the thoracotomy should be performed first. The interval between the two surgeries should not be too long. It has been reported that after the resection of the cardiac tumor, the tumor was found to enter the heart again during the second-stage surgery [29, 30]. In this study, except for one case of preoperative misdiagnosis as atrial myxoma and performed staging surgery, the other patients underwent one-stage surgical resection.

Currently, no more than 10 cases of right intracardiac tumor have been completely resected through abdominal incision [11, 12]. They believed that the resection of the tumor through abdominal incision should meet the following requirements: (1) the tumor was free in the inferior vena cava and right atrium; (2) the maximum diameter of the tumor was smaller than the minimum diameter of the inferior vena cava. In this study, we performed complete resection of tumors in right atrium, inferior vena cava, iliac vein, and pelvic cavity through single abdominal incision in 3 cases. The maximum diameter of the tumor in the three cases of abdominal resection exceeded the minimum diameter of the inferior vena cava. Besides, in Case 4, we removed the tumor in the right atrium and the inferior vena cava through the right ovarian vein, and the tumor was larger than the maximum diameter of the ovarian vein. The reason why we did this in Case 4 is that the tumor and the wall of the inferior vena cava are elastic. When the tension is encountered during traction, the tumor diameter shrinks, while the inferior vena cava expands. As long as there is no adhesion, the tumor will not encounter great resistance. Because the tumor originated from the internal iliac vein or ovarian vein, fixed with pelvic lesions, simple removal of abdominal tumor through chest incision is not desirable. There have been previous cases of renal vein rupture hemorrhage and death caused by tumor in inferior vena cava pulled through the heart [1, 13]. However, there have been no reports of pulmonary embolism and rupture of inferior vena cava caused by abdominal traction. We believe that this method has the following advantages: (1) no need for chest incision, more minimally invasive; (2) avoid coagulopathy and neurological sequelae caused by deep hypothermic circulatory arrest; (3) the tracheal intubation can be directly removed after surgery, without needing to go to ICU for monitoring; (4) reduced the operation time, hospital stay, and reduced costs.

As soon as IVL was diagnosed, surgery was required as soon as possible. The patients who died during the operation had long-term dyspnea and presented orthopnea at 1 week before the operation. According to the intraoperative situation, she died due to cardiogenic shock probably and had nothing to do with the operation itself. Intraoperatively, the tumor occupied the right atrium, right ventricle, main pulmonary artery, and bipulmonary artery. We found for the first time that the tumor extended upward from the right atrium into the superior vena cava, without previous reports of superior vena cava involvement.

Uterine fibroids are hormone-dependent tumor. Uterine fibroids in most patients with pausimenia stop growing or even shrink. While IVL originates from uterine fibroids, and there are many literatures that confirm the expression of ER and PR in intracardiac tumors [7, 31]. We have 5 cases of intracardiac tumor specimens expressing ER/PR to varying degrees. In theory, antiestrogen therapy is effective for IVL. There have also been reports of tamoxifen, gonadotropin releasing hormone analogs, and antiestrogen therapy [32, 33], but none of them can reduce postoperative tumor recurrence. In our opinion, tumor recurrence is not closely related to estrogen level, because two patients in this group still have recurrence after hysterectomy + bilateral adnexectomy. Moreover, Case 5 had a history of uterine fibroids for more than 10 years. The diameter of uterine fibroids decreased from 11 cm to 8 cm after menopause, but the tumor in blood vessels continued to grow and reached the heart. This may be because the growth environment of the intravenous leiomyoma was different from that of the uterus and its sensitivity to estrogen was different. Moreover, antiestrogen therapy has many side effects. Together, antiestrogen therapy is not recommended for patients with complete resection.

A total of 5 patients in this study developed deep vein thrombosis, with an incidence of 35.7% (5/14), while the incidence of thrombosis in patients with stage II or above was as high as 71.4% (5/7). There have been no previous reports of venous thrombosis after IVL surgery, which may be because for intracardiac leiomyomatosis, collateral circulation has long been established, and postoperative deep venous thrombosis of lower limbs is usually asymptomatic. In addition, most previous literatures were reported in cardiac surgery or gynecology, which were not concerned about thrombosis. Case 1, Case 3, and Case 4 were all found to have iliac vein/inferior vena cava thrombosis and pulmonary embolism by postoperative CT reexamination without clinical symptoms. We thought that the causes of thrombosis were as follows: (1) the operation needs to open the inferior vena cava or the iliac vein to remove the tumor, that is, the vascular injury; (2) prolonged bed immobilization after surgery leads to slow blood flow and high coagulation; (3) iliac vein compression (Figure 3(e)). These three reasons are in line with the three elements of Virchow thrombosis [34]. ICL is high risk with thrombosis, which requires routine anticoagulation treatment in the perioperative period, and the anticoagulation treatment course is recommended to be extended to 3 months after surgery.

In addition to the simple abdominal resection of the tumor in the right atrium to avoid thoracotomy, this study had the following advantages: (1) the first report of the inferior vena cava filter to protect the descending vena cava leiomyomas resection and the filter was removed after surgery; (2) IVL involvement in the superior vena cava was first reported; (3) the first report on thrombosis in IVL; (4) we divided the tumor into four types, and the fourth type has not been reported in the past; (5) the first report that retroperitoneal tumors entered the inferior vena cava and right atrium through renal veins. Since postoperative recurrence of IVL may occur after many years of surgery, long-term follow-up is necessary, and annual CT examination is recommended for patients with stage II or above. However, this is only a single-center retrospective study and the sample size is not large enough.

In conclusion, the onset of IVL is insidious and difficult to diagnose in the early stage. Once the disease is diagnosed, radical resection should be performed as soon as possible. The scope of surgical resection should include uterus and bilateral adnexectomy including all tumors. Incomplete or delayed resection can lead to serious complications such as recurrence or death. For IVL involving the right atrium, the tumor has no adhesion to the right atrium and the inferior vena cava. The relationship between the tumor and the inferior vena cava/right atrium can be confirmed by cardiac color Doppler ultrasound and enhanced CT examination, and the tumor type can be roughly judged. For the first type of tumor, the right heart tumor can be removed through the abdominal incision to avoid thoracotomy. If it is the second or third type of tumor, it can be tried to be pulled out from the inferior vena cava. Moreover, when resistance was encountered, the tumor should be removed from the heart in time using the thoracotomy. Inferior vena cava/right heart leiomyomatosis is at high risk of venous thrombosis, requiring routine anticoagulant treatment in the perioperative period, and the recommended anticoagulant course of treatment is up to 3 months after surgery.

## Figures and Tables

**Table 1:** Clinical data of patients with uterine intravenous leiomyomatosis.

Case	Age	Clinical feature	Extent of disease	Medical history	Surgical options	Follow-up
1	47	Dysuria and lower leg edema	Inferior vena cava	TAH-BSO at 3 years ago	Pelvic tumor/inferior vena cava tumor resection	Left iliac vein thrombosis
2	36	Pelvic cavity tumor	Right atrium	Hysteromyoma was resected for 2 years	TAH-BSO/radical surgery	AND
3	48	Chest distress and palpitation	Right atrium	Right atrial tumor was resected for 1 year	TAH-BSO/radical surgery	Left iliac vein thrombosis
4	61	Chest distress	Right atrium	Uterine fibroids for 10 years	TAH-BSO/radical surgery and gastrectomy	Pulmonary embolism/right ovarian vein stump thrombosis
5	43	Weakness and fainting	Pulmonary artery	—	Staging operation	Extensive thrombosis of the deep vein in the right lower extremity
6	66	Chest distress	Pulmonary artery/superior vena cava	—	Right heart tumor/pulmonary artery tumor resection (intraoperative death)	Intraoperative death
7	43	Abdominal pain	Right atrium	TAH at 8 months ago	Go to other hospitals for radical surgery	AND
8	35	Menstrual increase	Pelvic cavity	Uterine fibroids for 4 years	Hysteromyomectomy	Uterine fibroids recurred four years after surgery
9	44	Lower abdominal bulge	Pelvic cavity	—	TAH-BSO	AND
10	41	—	Pelvic cavity	—	TAH-BSO	AND
11	50	—	Pelvic cavity	—	TAH-BSO	AND
12	55	—	Pelvic cavity	—	Hysteromyomectomy	AND
13	39	Dysmenorrhea	Pelvic cavity	—	TAH-BSO	AND
14	33	Menstrual increase	Pelvic cavity	—	Hysteromyomectomy	AND

TAH-BSO: Total abdominal hysterectomy-Bilateral salpingo-oophorectomy. AND: alive with no evidence disease.

**Table 2:** The clinical data of patients with tumors in the inferior vena cava.

Case	Preoperative thrombosis	Operation durations (min)	Blood losses (ml)	Intraoperative blood transfusion	Anticoagulant solution
1	Medium risk	225	1600	Red blood cell 4 u Plasma 200 ml	12 hours after surgery, LMWH 5000 u ih qd
2	Medium risk	430	2500	Red blood cell 10 u Plasma 1000 ml	24 h after surgery, LMWH 5000 u ih qd
3	Medium risk	480	2000	Red blood cell 8 u Plasma 800 ml	12 hours after surgery, LMWH 5000 u ih q12 h
4	High risk	250	1200	Red blood cell 4 u	24 h after surgery, LMWH 5000 u ih q12 h
5 (first surgery)	High risk	215	500	No blood transfusion	12 hours after surgery, LMWH 5000 u ih q12 h
5 (secondary surgery)	High risk	355	2500	Red blood cell 12 u Plasma 400 ml	12 hours after surgery, LMWH 5000 u ih q12 h
6	High risk	195	1200	Red blood cell 4 u	Intraoperative death

LMWH: low molecular weight heparin.

## Data Availability

The raw data supporting the conclusions of this study will be made available by contacting the corresponding author.

## Additional Points

*Highlights.* (1) Once IVL is diagnosed, radical resection should be performed as soon as possible. (2) Specific surgical plans can be formulated according to cardiac ultrasound and CT. (3) Anticoagulant therapy is recommended until 3 months after surgery.

## Supplementary Materials

Supplementary Materials Supplementary Figure 1: a retroperitoneal tumor. A. the tumor enters the inferior vena cava to the right atrium via the left renal vein. B. right atrial tumor. C. the arrow indicates the left renal vein. Supplementary Table 1: changes in cardiac function before and after surgery and immunohistochemistry results of tumor in the inferior vena cava/right atrium.
