# Supplementary material for: Intravenous Leiomyomatosis of the Uterus: A Retrospective Single-Center Study in 14 Cases
Source: Biomed Res Int. 2020 Feb 14;2020:9758302. doi: 10.1155/2020/9758302 (PMC7155762; doi:10.1155/2020/9758302)
Supplement: Supplementary Materials — Supplementary Figure 1: a retroperitoneal tumor. A. the tumor enters the inferior vena cava to the right atrium via the left renal vein. B. right atrial tumor. C. the arrow indicates the left renal vein. Supplementary Table 1: changes in cardiac function before and after surgery and immunohistochemistry results of tumor in the inferior vena cava/right atrium. [file 9758302.f1.docx]

**Supplementary Figure 1.** A retroperitoneal tumor that entered the inferior vena cava and reached the right atrium via the left renal vein. A, the retroperitoneal tumor enters the inferior vena cava to the right atrium via the left renal vein. B, right atrial tumor. C, the arrow indicates the left renal vein.


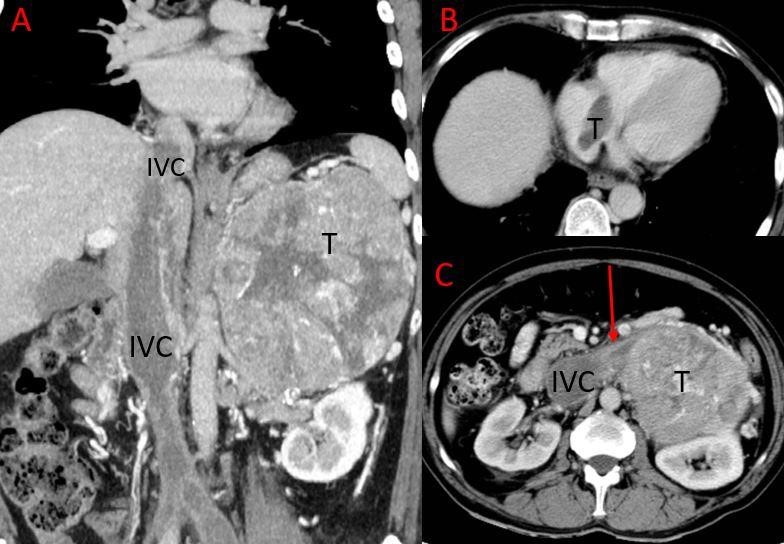


**Supplementary Table 1** Changes in cardiac function before and after surgery and immunohistochemistry results of tumor in the inferior vena cava/ right atrium

Case Heart function class（NYHA） Desmin SMA ER PR

| Pre-operation | Post-operation |  |  |  |  |
| --- | --- | --- | --- | --- | --- |
| 1 I | I | + | + |  |  |
| 2 II | I | + | + | + | + |
| 3 III | I | + | + | ++ | ++ |
| 4 III | II | + | + | +++ | +++ |
| 5 III | II | + | + | + | + |
| 6 IV | Death | + | + | + | - |
| 7 II | I |  |  |  |  |

SMA, smooth muscle actin; ER, oestrogen receptor; PR, progesterone receptor; NYHA, New York Heart Association
